# Socioeconomic inequalities in frailty and frailty components among community-dwelling older citizens

**DOI:** 10.1371/journal.pone.0187946

**Published:** 2017-11-09

**Authors:** Carmen B. Franse, Amy van Grieken, Li Qin, René J. F. Melis, Judith A. C. Rietjens, Hein Raat

**Affiliations:** 1 Department of Public Health, Erasmus University Medical Center, Rotterdam, The Netherlands; 2 Department of Geriatric Medicine, Radboud University Medical Center, Nijmegen, The Netherlands; Universita degli Studi di Napoli Federico II, ITALY

## Abstract

**Background:**

So far, it has not yet been studied whether socioeconomic status is associated with distinct frailty components and for which frailty component this association is the strongest. We aimed to examine the association between socioeconomic status and frailty and frailty components. In addition we assessed the mediating effect of the number of morbidities on the association between socioeconomic status and other frailty components.

**Methods:**

This is a cross-sectional study of pooled data of The Older Persons and Informal Caregivers Survey Minimum DataSet in the Netherlands among community-dwelling persons aged 55 years and older (n = 26,014). Frailty was measured with a validated Frailty Index that consisted of 45 items. The Frailty Index contained six components: morbidities, limitations in activities of daily living (ADL), limitations in instrumental ADL (IADL), health-related quality of life, psychosocial health and self-rated health. Socioeconomic indicators used were education level and neighbourhood socioeconomic status.

**Results:**

Persons with primary or secondary education had higher overall frailty and frailty component scores compared to persons with tertiary education (P < .001). Lower education levels were most consistently associated with higher overall frailty, more morbidities and worse self-rated health (P < .05 in all age groups). The strongest association was found between primary education and low psychosocial health for persons aged 55–69 years and more IADL limitations for persons aged 80+ years. Associations between neighborhood socioeconomic status and frailty (components) also showed inequalities, although less strong. The number of morbidities moderately to strongly mediated the association between socioeconomic indicators and other frailty components.

**Conclusion:**

There are socioeconomic inequalities in frailty and frailty components. Inequalities in frailty, number of morbidities and self-rated health are most consistent across age groups. The number of morbidities a person has play an important role in explaining socioeconomic inequalities in frailty and should be taken into account in the management of frailty.

## Introduction

Frailty can be defined as a state of increased vulnerability to external stressors and adverse outcomes such as death and hospitalization[1, 2]. Frailty is a better predictor of adverse outcomes than age[3]. Hence, it is important to identify persons or groups at risk of developing frailty in order to target prevention strategies. Older persons with a low socioeconomic status (SES) are more frail and become more frail over time compared to persons with a high SES[4–6]. Many indicators of SES such as education level, occupation, income and wealth have been linked to frailty[4, 7–9].

A widely used approach to measure frailty is the accumulation-of-deficits approach that results in a Frailty Index (FI)[10, 11]. The FI is calculated by adding up the number of health deficits a person has, divided by the total of possible health deficits included in the index. Theou et al. found that of eight commonly use frailty scales, the FI most accurately predicted mortality[12]. A standard procedure to construct a FI was developed by Searle et al., who recommended to include the following components in the index: morbidities, disability in Activities of Daily Living (ADL) and Instrumental ADL (IADL), restricted activity, impairments in general cognition and physical performance, psychological health and self-rated health (SRH)[13]. In addition to the study of ‘overall’ frailty, the assessment of frailty components could uncover important information about the specific domain in which a person is frail. Recently, Yang et al. have studied the associations of frailty components with mortality and found that IADL and ADL limitations played a greater role in mortality compared to other components[14].

It is not yet studied which frailty component contributes most strongly to socioeconomic inequalities in frailty. By uncovering this, interventions could be directed towards narrowing the gap in frailty between persons with a higher versus a lower SES. In the FI approach and other frailty measures such as the FRAIL scale, morbidities are considered as part of frailty[2, 12]. Theoretically morbidities precede the other frailty components of the FI, as proposed in different health models[15, 16]. Having certain morbidities at a younger age, such as depression or cardiovascular disease, could lead to an increase in ADL and/or IADL limitations at older age[17]. Studies using Fred’s frailty phenotype have showed that both number and specific morbidities such as obesity partly explained why persons with a lower SES were more frail compared to persons with a higher SES[9, 18]. Therefore we hypothesize that the presence of morbidities could mediate the association between SES and other components of the FI.

The aim of this study was, 1) to assess the association between SES indicators and a) ‘overall’ frailty and b) the distinct frailty components (morbidities, ADL, IADL, health-related quality of life (HRQoL), psychosocial health and SRH), and 2) to assess whether and to what extent the number of morbidities mediates the association between SES and the other frailty components (ADL, IADL, HRQoL, psychosocial health and SRH).

## Methods

### Study design and population

We applied a cross-sectional study design using data from The Older Persons and Informal Caregivers Survey Minimum DataSet (TOPICS-MDS)[19]. TOPICS-MDS is a data-base designed to capture information on the well-being of older persons in the Netherlands. TOPICS-MDS was developed to collect uniform information from studies funded under the National Care for older citizens Programme[20]. Included survey items were based on the recommendations of experts who identified key outcomes in older persons’ health[19]. Data were collected between 2010 and 2013 in 50 studies in the Netherlands. TOPICS-MDS consists of pooled data of these studies which differ across study design, sampling framework, and inclusion criteria. TOPICS-MDS is a fully anonymized data set, and therefore this analysis was exempt from ethical review (Radboud University Medical Centre Ethical Committee review reference number: CMO: 2012/120).

**Fig 1:**
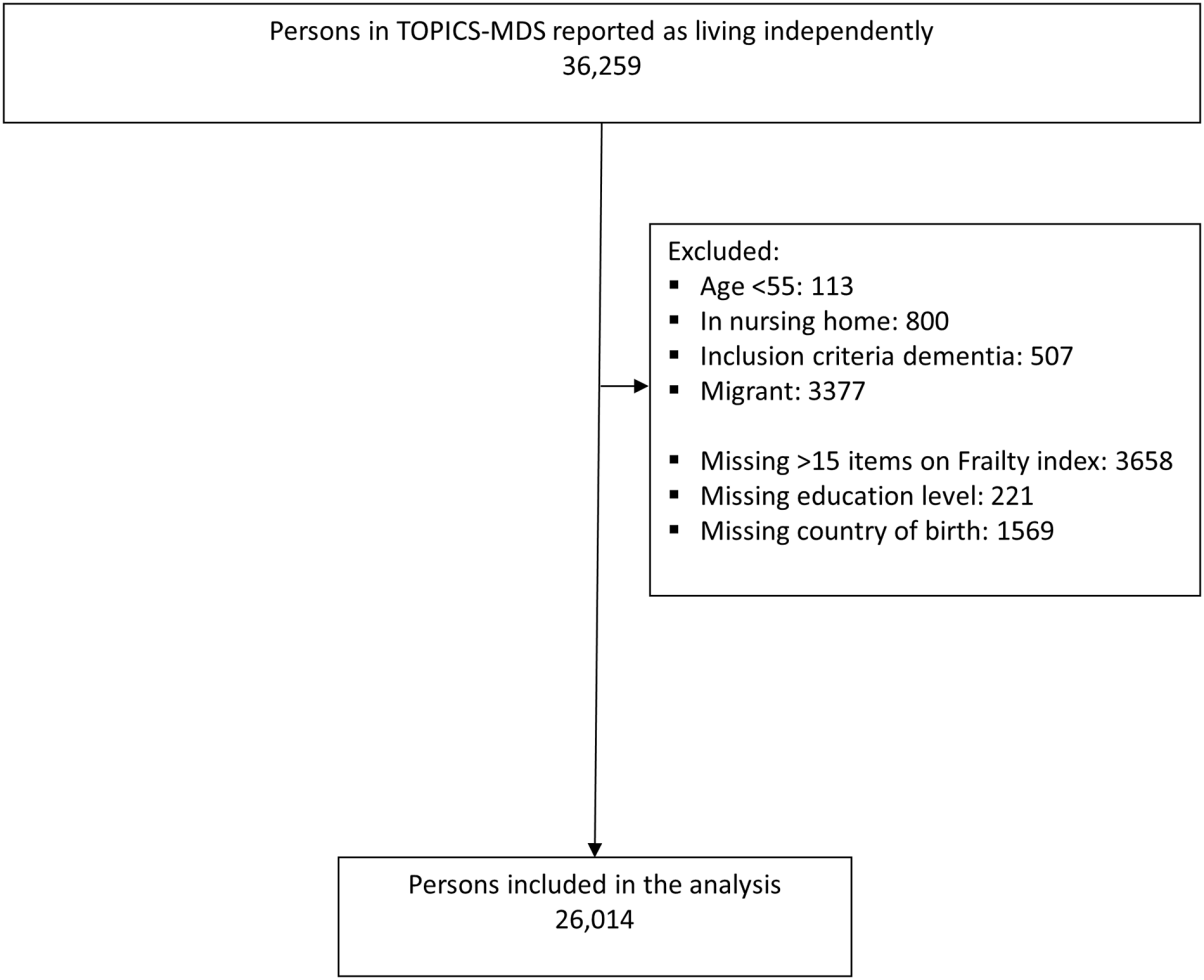
Population of analysis.

Our analysis was restricted to data from independently living Dutch persons aged 55 years and older. We further excluded persons with more than 15 missing items for the FI (n = 3658), missing education level (n = 221) or country of birth (n = 1569). The final sample comprised of data from 30 studies of 26,014 persons (see Fig 1).

### Frailty and components

Frailty was measured by the TOPICS-Frailty Index (TOPICS-FI), which was developed and validated using TOPICS-MDS data by Lutomski et al.[21]. In our study we included the 45 item TOPICS-FI, after exclusion of the item measuring prostatism. Searle et al. showed that a FI with 30–40 variables is accurate for predicting adverse outcomes[13, 22]. The TOPICS-FI was calculated when at least 30 items were available. This was done by adding up the number of health deficits a person reported, divided by the total health deficits measured for this person, following Searle et al.[13]. This resulted in a score between 0–1, where higher scores represent higher frailty.

The TOPICS-FI as used in this study consists of 45 items that belong to six components, each measured by validated instruments; morbidities, ADL, IADL, HRQoL, psychosocial health and SRH[13]. The component ‘Morbidities’ was measured by 16 items regarding the self-reported presence (yes/no) of diabetes, stroke, heart failure, cancer, respiratory condition (asthma, chronic bronchitis, lung emphysema or Chronic obstructive pulmonary disease (COPD), incontinence, joint damage of hips or knees, osteoporosis, hip fracture, fractures other than hip, dizziness with falling, depression, anxiety/panic disorder, dementia, hearing problems, vision problems. The component ‘ADL limitations’ was measured by 6 items using a modified version of the Katz instrument[23, 24]. Persons could indicate whether they needed help (yes/no) with the following activities: bathing, dressing, toileting, incontinence, sitting down, eating. The component ‘IADL limitations’ was measured by 9 items using a modified version of the Katz instrument[23, 24]. Persons could indicate whether they needed help (yes/no) with the following activities: using the telephone, travelling, shopping, preparing a meal, cleaning, taking medications, handling finance, brushing hair and walking. The component ‘HRQoL’ was measured by 6 items of the EuroQol 5D+C[25]. Persons could indicate whether they had problems (no/some/extreme) with the following: mobility, self-care, usual activities, pain/discomfort, anxiety/depression and cognition. The component ‘Psychosocial health’ was measured with 5 items of the RAND-36 [26]. Persons could indicate how much of the time in the past month (none/a little/ some/a good bit/most/all) they had been the following: nervous, calm, downhearted, happy and down in the dumps, and how much time (none/a little/some/most/all) health problems had interfered with social activities. The component ‘SRH’ was measured with 2 items of the RAND-36[26], one regarding perceived current health status (poor/fair/good/very good/excellent) and one regarding perceived changes in health in the past year (much worse/slightly worse/about the same/a little better/much better). The score for each component of the TOPICS-FI were calculated analogous to the FI, by adding up the health deficits within the FI component that a person had, divided by the total of possible health deficits included in the component[13]. This resulted in a score between 0–1, where higher scores represent worse health. We accepted no missing variables for SRH and a maximum of 1 of 3 missing variables for other FI component scores.

### Indicators of SES

In this study we applied two indicators of SES; education level and neighbourhood SES. TOPICS-MDS used the 1997 International Standard Classification of Education[27] to assess education level; participants were asked whether they had completed: fewer than 6 years of primary school; 6 years of primary school; further uncompleted education; vocational school; secondary professional education or university entrance level or tertiary education. We categorized the education level into “primary education or less”, “secondary education” and “tertiary education or higher”, based on the definition by Statistics Netherlands[28].

For the neighbourhood SES, the 2006 reference scores for area codes were used, as calculated by The Netherlands Institute for Social Research[29] based on the education level, income and labor market position of persons living in each area code. Scores were categorized into quartiles, quartile 1 is the least deprived quartile (high education, high income, high labor market position), while quartile 4 is the most deprived.

### Potential confounders

Gender, age, living arrangement, marital status and level of urbanization were incorporated as potential confounders in this study based on literature and availability in TOPICS-MDS. Age was assessed by asking year of birth. Living arrangement was assessed by asking whether participants were living: independent alone, independent with others, care or nursing home. Only persons living independently were included and categorized into “not alone” and “alone”. Marital status was assessed by asking whether participants were: married, divorced, widowed, unmarried, long term cohabitation unmarried. Answers were categorized into “married/cohabitant partners”, “divorced”, “widowed” and “single”. Level of urbanization was based on the density of addresses in an area code and categorized as by Statistics Netherlands into “not urban”, “little urban”, “somewhat urban”, “urban” and “very urban”[30].

### Statistical analysis

The statistical significance of differences in socio-demographic characteristics, frailty and frailty components (morbidities, ADL limitations, IADL limitations, psychosocial health, HRQoL and SRH) among persons from different education levels was calculated using chi-squared tests for categorical variables and one-way ANOVA for continues variables.

**Fig 2:**
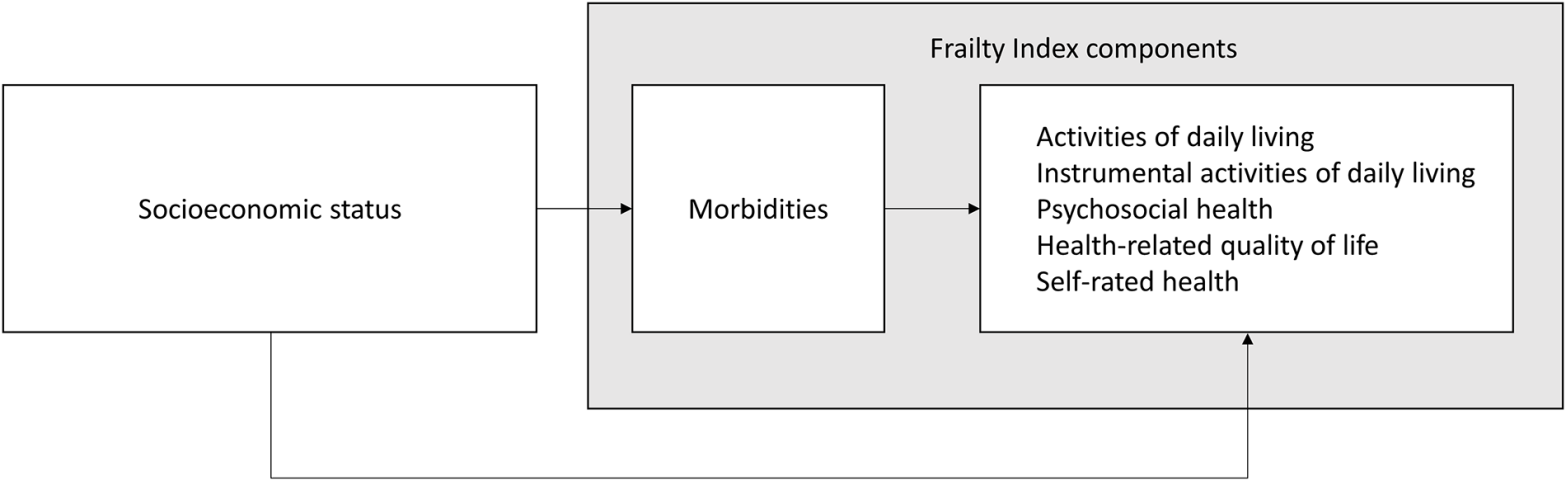
Conceptual framework for the association between socioeconomic status and Frailty Index components, where the morbidities component mediates the association between socioeconomic status and other Frailty Index components.

To examine the association between SES, frailty and frailty components (Model 1), we estimated multilevel random-intercept models because data were clustered in studies[31]. As such, dependency between the observations of participants of a study because of sampling design and/or inclusion criteria, was taken into account. Only potential confounders that led to a substantial change in effect estimates (i.e. ≥10% change) were included in models[32]. Subsequently, we examined the presence of mediation by the number of morbidities in the association between SES and other frailty components, by following the causal step approach proposed by Baron and Kenny (Fig 2)[33]. When SES indicators were significantly associated with the morbidities component and when the morbidities component was significantly associated with the other frailty components, the morbidities component was considered a ‘true’ mediator. Only then, the morbidities component was added to Model 1 (Model 2). To assess the mediating effect, the percentages of attenuation of effect estimates were calculated by comparing Model 2 relative to Model 1.

We explored the presence of interaction between the indicators of SES and sex, age and living arrangement in the association between SES and frailty and frailty components. We also explored interaction between the indicators of SES and morbidities (exposure-mediator interaction) in the association between SES and frailty and frailty components. After applying Bonferroni correction for multiple testing[34], we found significant interactions between SES and age on overall frailty and on all frailty components, and therefore stratified all analyses by age in three groups: 55–69 years, 70–79 years, and 80 years and older.

Percentages of missing values in the potential confounders were 2% or less (Table 1). Missing data on potential confounders were imputed using multiple imputation. We computed five imputation datasets using a fully conditional specified model[35]. Pooled estimates from these datasets were used to report regression coefficients and 95% confidence intervals (CIs). We considered a p-value of .05 or lower to be statistically significant for main analyses and used Bonferroni correction for testing interactions[34]. Descriptive analyses were performed using SPSS version 23.0 (IBM SPSS Statistics for Windows, Armonk, NY: IBM Corp). Multilevel linear regression analysis were performed using R-3.3.2.

**Table 1:** Socio-demographic characteristics and frailty outcomes by education level of 26,014 persons of The Older Persons and Informal Caregivers Survey Minimum DataSet (TOPICS-MDS).

	Total N = 26,014	Education level			*P*-value*
		Tertiary N = 2723	Secondary N = 14,762	Primary or less N = 8529	
Age in years (mean, SD)	78.0 (6.8)	76.1 (7.1)	77.7 (6.7)	79.3 (6.7)	<0.001
Sex, N (%)					<0.001
Male	10,825 (41.6)	1800 (66.1)	6394 (43.3)	2631 (30.8)	
Female	15,189 (58.4)	923 (33.9)	8268(56.7)	5898 (69.2)	
Living arrangement, N (%)					<0.001
Alone	11,689 (44.9)	860 (31.6)	6377 (43.2)	4452 (52.2)	
With others	14,325 (55.1)	1863 (68.4)	8385 (56.8)	4077 (47.8)	
Marital status, N (%)					<0.001
Married/Cohabitant partners	13,954 (53.6)	1836 (67.4)	8261 (56.0)	3857 (45.2)	
Divorced	1562 (6.0)	189 (7.0)	878 (5.9)	494 (5.8)	
Widowed	9288 (35.7)	491 (18.0)	4940 (33.5)	3857 (45.2)	
Single	1211 (4.7)	206 (7.6)	684 (4.6)	321 (3.8)	
Neighbourhood SES, N (%)					<0.001
First quartile	7277 (28.5)	1298 (48.2)	4369 (30.1)	1610 (19.4)	
Second quartile	6988 (27.4)	649 (24.1)	4012 (27.7)	2327 (28.0)	
Third quartile	5259 (20.6)	427 (15.9)	2958 (20.4)	1874 (22.6)	
Fourth quartile	5970 (23.4)	320 (11.9)	3165 (21.8)	2485 (30.0)	
Level of urbanization, N (%)					<0.001
Not urban	5802 (22.3)	592 (21.7)	3232 (21.9)	1978 (23.2)	
Little urban	7031 (27.0)	578 (21.2)	4177 (28.3)	2277 (26.7)	
Somewhat urban	4114 (15.8)	637 (23.4)	2410 (16.3)	1067 (12.5)	
Urban	6313 (24.3)	704 (25.9)	3497 (23.7)	2112 (24.8)	
Very urban	2754 (10.6)	213 (7.8)	1445 (9.8)	1096 (12.8)	
Overall Frailty mean FI (SD)†	0.20 (0.12)	0.16 (0.11)	0.20 (0.12)	0.23 (0.13)	<0.001
Morbidities, mean FI (SD)†	0.17 (0.12)	0.14 (0.11)	0.16 (0.12)	0.18 (0.13)	<0.001
Number morbidities, mean (SD)	2.61 (1.90)	2.16 (1.69)	2.55 (1.87)	2.88 (1.98)	<0.001
ADL limitations, mean FI (SD)†	0.11 (0.19)	0.08 (0.17)	0.11 (0.19)	0.13 (0.20)	<0.001
Number ADL limitations, mean (SD)	0.65 (1.10)	0.47 (0.97)	0.62 (1.08)	0.78 (1.16)	<0.001
IADL limitations, mean FI (SD)†	0.21 (0.24)	0.14 (0.21)	0.20 (0.23)	0.26 (0.25)	<0.001
Number IADL limitations, mean (SD)	1.48 (1.67)	0.96 (1.47)	1.39 (1.62)	1.81 (1.74)	<0.001
Psychosocial health, mean FI (SD)†	0.26 (0.18)	0.22 (0.16)	0.25 (0.17)	0.28 (0.19)	<0.001
Health-related quality of life, mean FI (SD)†	0.22 (0.17)	0.18 (0.16)	0.21 (0.17)	0.25 (0.17)	<0.001
Self-rated Health, mean FI (SD)†	0.58 (0.17)	0.54 (0.17)	0.57 (0.17)	0.60 (0.17)	<0.001

* P-values are based on Chi-squared test for categorical variables and one-way ANOVA for continues variables.

^†^ Mean FI = mean number of health deficits reported/total health deficits measured in instrument; score between 0–1 where higher scores represent worse health. Missing N (%) for variables: Age = 544 (2%); sex = 8 (<1%); living arrangement = 0 (0%); marital status = 50 (<1%); Neighbourhood SES = 520 (2%); Level of urbanization = 199 (1%); morbidities = 531 (2%); ADL = 45 (<1%); IADL = 124 (<1%); psychosocial health = 281 (1%); Health-related quality of life = 521 (2%); Self-rated health = 100 (<1%). FI = frailty index; (I)ADL = (instrumental) activities of daily living; SES = socioeconomic status.

### Non-response analysis

A comparison of persons included in the study (N = 26,014) with persons not included due to missing values for education level, FI and/or country of birth (N = 5448) did not indicate significant differences in terms of sex (p = .882) and living arrangement (p = .113). However, excluded persons were older (p < .001), more often single (p < .05), more often living in rural areas and in deprived neighbourhoods (p < .001) than persons included in the study.

## Results

Table 1 shows the characteristics of the study population. Of all persons, 10.5% of the persons had tertiary education, and 32.8% had primary education or less. Compared with persons who received tertiary education, persons who received primary education or less were older, more often female, more often living alone, more often widowed and less often married, single or divorced and more often living in deprived neighbourhoods (P < .001). Frailty was highest in persons who received primary education or less (mean = 0.23; SD = 0.13), followed by persons who received secondary education (mean = 0.20; SD = 0.12) and persons who received tertiary education (mean = 0.16; SD = 0.11).

Education level was significantly associated with frailty; frailty was higher in persons of all age groups with secondary and primary or less education as compared to persons with tertiary education (p < .05; Table 2-Model 1). This was also found for the frailty components morbidities and SRH. Persons with lower education levels generally had higher scores (i.e. worse health) for IADL limitations, psychosocial health and HRQoL, although not significant in all age groups for secondary education. ADL limitations were only worse in persons aged 70–79 years with primary education or less compared to persons with tertiary education (p < .05). Among all frailty components, the association between education level and psychosocial health was strongest in persons aged 55–69 years, while for persons aged 80+ years this was IADL limitations. For frailty and all frailty components except IADL limitations, stronger associations were observed in persons aged 55–69 compared to older age groups. The number of morbidities mediated the association between education level and other frailty components, attenuations ranged between 19% and 80% (Table 2-Model 2).

**Table 2:** Association of education level with overall frailty and with its six components (Model 1) and change in association of education level with the five other frailty components after adjustment for the morbidities component (Model 2); stratified by age group among 26,014 persons of The Older Persons and Informal Caregivers Survey Minimum DataSet (TOPICS-MDS).

	Overall Frailty	Morbidities	ADL limitations		IADL limitations		Psychosocial health		Health-related quality of life		Self-rated health	
	B (95% CI)	B (95% CI)	B (95% CI)	%†	B (95% CI)	%†	B (95% CI)	%†	B (95% CI)	%†	B (95% CI)	%†
Model 1												
Age 55–69 years												
Secondary education	0.016**	0.019***	0.007		0.005		0.016		0.020*		0.028**	
	(0.005–0.026)	(0.008–0.030)	(-0.008–0.022)		(-0.013–0.023)		(-0.003–0.036)		(0.003–0.038)		(0.011–0.046)	
≤ Primary education	0.047***	0.052***	0.013		0.034**		0.074***		0.056***		0.064***	
	(0.035–0.059)	(0.039–0.064)	(-0.005–0.030)		(0.013–0.055)		(0.051–0.096)		(0.036–0.076)		(0.044–0.085)	
Age 70–79 years												
Secondary education	0.007*	0.008**	-0.002		0.011*		0.006		0.005		0.016***	
	(0.001–0.013)	(0.002–0.014)	(-0.010–0.007)		(0.000–0.021)		(-0.003–0.015)		(-0.004–0.014)		(0.007–0.025)	
≤ Primary education	0.027***	0.022***	0.010*		0.039***		0.034***		0.029***		0.043***	
	(0.021–0.033)	(0.015–0.028)	(0.000–0.019)		(0.028–0.051)		(0.024–0.044)		(0.019–0.038)		(0.033–0.053)	
Age ≥ 80 years												
Secondary education	0.015***	0.012**	0.001		0.019*		0.022***		0.018**		0.014*	
	(0.006–0.024)	(0.003–0.021)	(-0.014–0.016)		(0.001–0.036)		(0.009–0.035)		(0.005–0.031)		(0.001–0.026)	
≤ Primary education	0.026***	0.019***	0.012		0.044***		0.026***		0.033***		0.022***	
	(0.017–0.036)	(0.009–0.028)	(-0.003–0.028)		(0.026–0.063)		(0.012–0.040)		(0.019–0.046)		(0.007–0.035)	
Model 2												
Age 55–69 years												
Secondary education	NA	NA	0.001	NA	-0.003	NA	0.003	NA	0.009	55%	0.017*	39%
			(-0.014–0.16)		(-0.021–0.014)		(-0.015–0.022)		(-0.007–0.025)		(0.001–0.034)	
≤ Primary education	NA	NA	-0.003	NA	0.014	59%	0.040***	46%	0.026**	54%	0.036***	44%
			(-0.021–0.014)		(-0.007–0.034)		(0.019–0.062)		(0.007–0.044)		(0.016–0.056)	
Age 70–79 years												
Secondary education	NA	NA	-0.005	NA	0.006	45%	0.002	NA	0.001	NA	0.013**	9%
			(-0.014–0.004)		(-0.004–0.016)		(-0.007–0.011)		(-0.007–0.008)		(0.004–0.021)	
≤ Primary education	NA	NA	0.002	80%	0.028***	28%	0.024***	29%	0.016***	45%	0.032***	26%
			(-0.008–0.011)		(0.017–0.039)		(0.014–0.034)		(0.007–0.025)		(0.023–0.042)	
Age ≥ 80 years												
Secondary education	NA	NA	-0.005	NA	0.011	42%	0.016**	27%	0.010	44%	0.008	43%
			(-0.020–0.010)		(-0.006–0.028)		(0.003–0.028)		(-0.002–0.022)		(-0.004–0.020)	
≤ Primary education	NA	NA	0.003	NA	0.032***	27%	0.017**	35%	0.021***	36%	0.013*	41%
			(-0.012–0.019)		(0.015–0.050)		(0.003–0.030)		(0.008–0.033)		(0.001–0.025)	

Values are derived from multilevel multivariable linear regression, tertiary education is the reference group. Model 1 is adjusted for confounders: age, sex, and living arrangement (alone/not alone). Model 2 is additionally adjusted for morbidities.

* p<0.05;

** p<0.01;

*** p<0.001.

^†^ % represents the % change in effect estimates relative to model 1 after adjustment for morbidities; this was calculated by: 100x ((B_model1_-B_model2_)/B_model1_). B = effect estimate; CI = confidence interval; (I)ADL = (instrumental) activities of daily living

Neighbourhood SES was significantly associated with frailty, morbidities, IADL limitations, psychosocial health, HRQoL and SRH (p < .05; Table 3-Model 1). Persons living in more deprived neighbourhoods (third or fourth quartile) had higher scores compared to those living in the least deprived neighbourhoods (first quartile). The number of morbidities mediated the association between neighbourhood SES and other frailty components, attenuations ranged between 20% and 90% (Table 3-Model 2).

**Table 3:** Association of neighbourhood socioeconomic status with overall frailty and with its six components (Model 1) and change in association of neighbourhood socioeconomic status with the five other frailty components after adjustment for the morbidities component (Model 2); stratified by age group among 25,494 persons of The Older Persons and Informal Caregivers Survey Minimum DataSet (TOPICS-MDS).

	Overall Frailty	Morbidities	ADL limitations		IADL limitations		Psychosocial health		Health-related quality of life		Self-rated health	
	B (95% CI)	B (95% CI)	B (95% CI)	%†	B (95% CI)	%†	B (95% CI)	%†	B (95% CI)	%†	B (95% CI)	%†
Model 1												
Age 55–69 years												
Second quartile	0.008	0.006	0.005		-0.005		0.020		0.017		0.021*	
	(-0.004–0.020)	(-0.007–0.018)	(-0.012–0.022)		(-0.025–0.016)		(-0.002–0.042)		(-0.003–0.036)		(0.0011–0.041)	
Third quartile	0.012*	0.013	-0.004		0.005		0.023*		0.028**		0.021*	
	(0.001–0.023)	(-0.001–0.025)	(-0.021–0.012)		(-0.015–0.025)		(0.001–0.044)		(0.010–0.047)		(0.001–0.040)	
Fourth quartile	0.023***	0.025***	-0.002		0.016		0.043***		0.028**		0.036***	
	(0.012–0.033)	(0.014–0.036)	(-0.017–0.013)		(-0.002–0.034)		(0.024–0.063)		(0.011–0.045)		(0.018–0.053)	
Age 70–79 years												
Second quartile	0.003	0.007**	-0.001		0.000		0.001		0.006		0.002	
	(-0.001–0.008)	(0.002–0.012)	(-0.008–0.006)		(-0.009–0.009)		(-0.007–0.009)		(-0.001–0.013)		(-0.006–0.009)	
Third quartile	0.010***	0.012***	0.002		0.007		0.010*		0.013**		0.008	
	(0.004–0.015)	(0.006–0.018)	(-0.006–0.010)		(-0.003–0.017)		(0.001–0.018)		(0.005–0.022)		(-0.001–0.017)	
Fourth quartile	0.014***	0.015***	0.004		0.010*		0.016***		0.018***		0.025***	
	(0.008–0.019)	(0.009–0.021)	(-0.005–0.012)		(0.000–0.021)		(0.007–0.025)		(0.009–0.026)		(0.016–0.033)	
Age ≥ 80 years												
Second quartile	0.005	0.006	0.002		0.012		-0.003		0.010*		-0.002	
	(-0.001–0.011)	(-0.001–0.012)	(-0.008–0.013)		(-0.001–0.024)		(-0.012–0.007)		(0.001–0.019)		(-0.011–0.007)	
Third quartile	0.003	0.008*	-0.002		0.002		0.000		0.003		0.003	
	(-0.004–0.010)	(0.001–0.015)	(-0.014–0.010)		(-0.012–0.015)		(-0.011–0.010)		(-0.007–0.013)		(-0.007–0.013)	
Fourth quartile	0.012***	0.008*	0.004		0.017*		0.017**		0.025***		0.010*	
	(0.005–0.019)	(0.001–0.015)	(-0.008–0.016)		(0.003–0.030)		(0.006–0.027)		(0.015–0.035)		(0.000–0.020)	
Model 2												
Age 55–69 years												
Second quartile	NA	NA	0.003	NA	-0.007	NA	0.015	NA	0.014	NA	0.019*	NA
			(-0.014–0.20)		(-0.027–0.013)		(-0.006–0.036)		(-0.005–0.032)		(0.000–0.038)	
Third quartile	NA	NA	-0.007	NA	0.001	NA	0.015	NA	0.023**	NA	0.015	NA
			(-0.023–0.009)		(-0.019–0.020)		(-0.005–0.035)		(0.006–0.040)		(-0.003–0.033)	
Fourth quartile	NA	NA	-0.009	NA	0.005	NA	0.027**	37%	0.014	50%	0.022**	39%
			(-0.024–0.006)		(-0.013–0.022)		(0.008–0.045)		(-0.002–0.029)		(0.005–0.039)	
Age 70–79 years												
Second quartile	NA	NA	-0.004	NA	-0.004	NA	-0.003	NA	0.002	NA	-0.002	NA
			(-0.011–0.004)		(-0.013–0.004)		(-0.010–0.005)		(-0.004–0.009)		(-0.010–0.005)	
Third quartile	NA	NA	-0.003	NA	0.000	NA	0.003	70%	0.006	54%	0.000	NA
			(-0.011–0.005)		(-0.010–0.010)		(-0.006–0.011)		(-0.002–0.014)		(-0.008–0.008)	
Fourth quartile	NA	NA	-0.003	NA	0.001	90%	0.008	50%	0.008*	56%	0.016***	36%
			(-0.011–0.006)		(-0.008–0.011)		(-0.001–0.016)		(0.001–0.016)		(0.007–0.024)	
Age ≥ 80 years												
Second quartile	NA	NA	0.000	NA	0.008	NA	-0.006	NA	0.006	NA	-0.005	NA
			(-0.011–0.010)		(-0.004–0.020)		(0.015–0.003)		(-0.002–0.015)		(-0.014–0.003)	
Third quartile	NA	NA	-0.006	NA	-0.004	NA	-0.004	NA	-0.001	NA	-0.001	NA
			(-0.017–0.006)		(-0.017–0.010)		(-0.014–0.006)		(-0.010–0.008)		(-0.011–0.008)	
Fourth quartile	NA	NA	0.001	NA	0.012	29%	0.013**	24%	0.020***	20%	0.007	30%
			(-0.010–0.013)		(-0.001–0.026)		(0.003–0.023)		(0.011–0.030)		(-0.003–0.016)	

Values are derived from multilevel multivariable linear regression, First Quartile is the reference group. Model 1 is adjusted for confounders: age, sex, and living arrangement (alone/not alone). Model 2 is additionally adjusted for morbidities.

* p<0.05;

** p<0.01;

*** p<0.001.

^†^ % represents the % change in effect estimates relative to model 1 after adjustment for morbidities; this was calculated by: 100x ((B_model1_-B_model2_)/B_model1_). B = effect estimate; CI = confidence interval; (I)ADL = (instrumental) activities of daily living

## Discussion

This study showed that persons with the lowest SES, e.g. the lowest education level or living in the most deprived neighbourhoods, had the highest overall frailty and frailty component scores. The number of morbidities mediated the association between SES indicators and other frailty components.

In our study, education level was most consistently **associated** with overall frailty, morbidities and SRH. Former research found that lower educated persons are on average more frail compared to higher educated persons[5–8]. Education level has been associated with frailty components, such as ADL, IADL and SRH, although few studies compare multiple outcomes[36–38]. We found associations of neighborhood SES as indicator of individual SES with frailty and with frailty components, but these were generally less strong. Additionally, we examined the isolated effect of neighbourhood SES after adjustment for individual education level and found consistent associations for the most deprived neighbourhoods (S1 Table). Few studies have investigated the association between neighbourhood SES and frailty[39]. The association between the SES indicators and ADL limitations was not consistent in our study, which might be due to a ceiling effect for the instrument used in a community-dwelling population[40].

We found the strongest association between education level and psychosocial health for persons aged 55–69 years and with IADL limitations for persons aged 80 years and over. Vaughan et al. found that persons who had no cardiovascular disease when aged between 65–80 years maintained good physical functioning over the age of 80 years[17]. As certain morbidities are more prevalent among persons with a lower SES, this could at a younger age result in worse psychosocial health or self-rated health, but may as one ages increasingly impact on functional health[17, 41]. Socioeconomic inequalities in frailty and all frailty components except for IADL limitations, were larger among persons aged 55–69 years compared to older persons. This finding is often explained by a ‘healthy survivor effect’, where unhealthier persons with a low SES have died at a younger age and is found in cross-sectional research for various health outcomes[42–45]. However, longitudinal research has found confirming and contradicting results, depending on the indicator by which SES and health is measured[4, 46]. Further research is needed to understand the mechanisms behind these findings.

The number of morbidities moderately to strongly mediated the association between SES indicators and other frailty components. Former research has found that both specific morbidities and number of morbidities mediate socioeconomic inequalities in frailty[9, 18, 47, 48]. Hoogendijk et al. found that cognitive impairment, obesity, and number of chronic diseases had the largest contributions to socioeconomic inequalities in frailty[18], while Soler-Vila et al. found largest contributions for obesity, depression and musculoskeletal disease[9]. These studies have looked at physical frailty as developed by Fried[49]. A study by Gobbens et al. that used a multidimensional concept of frailty found that multi-morbidity could explain income differences in psychological and physical frailty, but not social frailty[47]. More longitudinal research is needed on the role of specific morbidities and number of morbidities in explaining socioeconomic inequalities in frailty and frailty components. Furthermore, this means that frailty and morbidities more often coexist in persons with a low SES. It is important to manage the progression of morbidities in this group, as the presence of frailty in persons with chronic diseases such as diabetes and COPD has shown to strongly increase mortality[50, 51].

The main strengths of this study are the size and diversity of the study population, this study included data from a large number of older persons from different regions in the Netherlands. Furthermore, we used validated instruments to measure frailty and frailty components. This study has some limitations. First, this study had a cross-sectional design, which limits conclusions regarding causality. Health could also impact a person’s SES, which is defined as health selection, however the effect of health selection is small for education level[52]. Second, there was considerable variation between the 30 included projects regarding sampling frame, inclusion criteria, study design, sample size, and data collection method. We used meta-analyses techniques to correct for clustering between subjects in projects. However, we believe that these pooled data are likely to reflect reality better than data from a single project based on one nonrandom sample. Third, due to item non-response there were some missing data. To deal with this we used multiple imputation methods for potential confounders. A non-response analysis showed that there were some socio-demographic differences between persons who were excluded and who were included, although it is unclear how this could have affected the size of the effect. We additionally performed a series of sensitivity analyses restricted to persons who had a complete number of items for the FI and for each FI component, changes were marginal.

In conclusion, there are socioeconomic inequalities in frailty and frailty components. Inequalities in frailty, number of morbidities and SRH are most consistent across age groups. The number of morbidities a person has play a role in explaining socioeconomic inequalities in frailty and should be considered in the management of frailty.

## Supporting information

S1 Table Association of neighbourhood socioeconomic status with frailty by age group and with frailty components by age group, corrected for individual education level, among 25,494 persons of The Older Persons and Informal Caregivers Survey Minimum DataSet (TOPICS-MDS). (DOCX)
