# Supplementary material for: Socioeconomic inequalities in frailty and frailty components among community-dwelling older citizens
Source: PLoS One. 2017 Nov 9;12(11):e0187946. doi: 10.1371/journal.pone.0187946 (PMC5679620; doi:10.1371/journal.pone.0187946)
Supplement: S1 Table — (DOCX) [file pone.0187946.s001.docx]

| Table S2. Association of neighbourhood socioeconomic status with frailty by age group and with frailty components by age group , corrected for individual education level, among 25,494 persons of The Older Persons and Informal Caregivers Survey Minimum DataSet (TOPICS-MDS). | | | | | | | | | | | | | |  |
| --- | --- | --- | --- | --- | --- | --- | --- | --- | --- | --- | --- | --- | --- | --- |
|  | Frailty Index | | Morbidities | | ADL  limitations | | IADL limitations | | Psychosocial health | | Health-related quality of life | | Self-rated health | |
|  | B (95% CI) | | B (95% CI) | | B (95% CI) | | B (95% CI) | | B (95% CI) | | B (95% CI) | | B (95% CI) | |
| Age 55-69 years |  | |  | |  | |  | |  | |  | |  | |
| Second quartile | 0.004  (-0.008-0.016) | | 0.001  (-0.012-0.014) | | 0.003  (-0.014-0.020) | | -0.007  (-0.028-0.013) | | 0.014  (-0.008-0.036) | | 0.012  (-0.008-0.031) | | 0.016  (-0.004-0.036) | |
| Third  quartile | 0.007  (-0.005-0.018) | | 0.007  (-0.005-0.019) | | -0.006  (-0.023-0.010) | | 0.001  (-0.019-0.021) | | 0.014  (-0.007-0.035) | | 0.022*  (0.003-0.040) | | 0.014  (-0.005-0.033) | |
| Fourth quartile | 0.014**  (0.003-0.024) | | 0.015**  (0.004-0.026) | | -0.005  (-0.020-0.011) | | 0.009  (-0.009-0.028) | | 0.030**  (0.010-0.050) | | 0.018*  (0.000-0.035) | | 0.024**  (0.006-0.041) | |
| Age 70-79 years | |  | |  | |  | |  | |  | |  | | |
| Second quartile | 0.002  (-0.003-0.007) | | 0.005*  (0.000-0.012) | | -0.002  (-0.009-0.006) | | -0.002  (-0.011-0.006) | | -0.001  (-0.009-0.007) | | 0.004  (-0.003-0.012) | | -0.001  (-0.009-0.007) | |
| Third  quartile | 0.008**  (0.002-0.013) | | 0.010***  (0.005-0.016) | | 0.001  (-0.007-0.009) | | 0.001  (-0.007-0.009) | | 0.007  (-0.002-0.016) | | 0.011**  (0.003-0.019) | | 0.004  (-0.004-0.013) | |
| Fourth quartile | 0.011***  (0.005-0.016) | | 0.013***  (0.007-0.018) | | 0.002  (-0.006-0.011) | | 0.002  (-0.006-0.011) | | 0.012**  (0.003-0.020) | | 0.014***  (0.006-0.023) | | 0.019***  (0.011-0.028) | |
| Age ≥ 80 years | |  | |  | |  | |  | |  | |  | | |
| Second quartile | 0.002  (-0.004-0.009) | | 0.004  (-0.002-0.011) | | 0.000  (-0.011-0.011) | | 0.006  (-0.006-0.018) | | -0.005  (-0.014-0.005) | | 0.007  (-0.003-0.016) | | -0.004  (-0.013-0.005) | |
| Third  quartile | 0.001  (-0.006-0.008) | | 0.007  (-0.001-0.014) | | -0.004  (-0.016-0.008) | | -0.003  (-0.017-0.011) | | -0.002  (-0.012-0.009) | | 0.000  (-0.010-0.011) | | 0.001  (-0.009-0.011) | |
| Fourth quartile | 0.009*  (0.002-0.016) | | 0.006  (-0.001-0.013) | | 0.002  (-0.010-0.014) | | 0.011  (-0.003-0.024) | | 0.014**  (0.004-0.024) | | 0.021**  (0.011-0.031) | | 0.007  (-0.003-0.017) | |
| Values are derived from multilevel multivariable linear regression, First Quartile is the reference group. Model is adjusted for: age, sex, living arrangement (alone/not alone) and education level.  * p<0.05; ** p<0.01; ** p<0.001.  B=effect estimate; CI=confidence interval; (I)ADL=(instrumental) activities of daily living. | | | | | | | | | | | | | |  |
